# Miscellaneous Notes and Comments

**Published:** 1914-03-14

**Authors:** 


### NATIONAL INSURANCE ACT DEVELOPMENTS.

### Miscellaneous Notes and Comments.

An Inter-Departmental Committee of the House of Commons, presided over by Sir Thomas Elliott, has recently been considering the question of the payment of benefits under the Insurance Act otherwise than in cash. The Committee presented a report to the Treasury, which was issued on February 10 [Cd. 7,245], and suggest that a special form of money order should be printed approximately the size of a postal order, and that these orders should be bound together in books, each containing 50 or 100 orders. It is suggested that a typical order would read:—

"To the Postmaster-General. Pay to ———, No. ———, the sum of ten shillings in respect of ——— (benefit under the National Insurance Act) ———for the period from ——— to ———. For the Approved Society." The order would, of course, be signed by the persons authorising payment, and also by the payee.

These orders would be payable at any post office, but the Committee proposes that they should become void unless cashed within one month of the date of issue. Payment would be made without reference to the amount standing to the credit of the approved society, but the issue of orders to the latter would be subject to the Commissioners' approval. It is estimated that the cost of printing and other expenses will be rather more than 1d. an order, and the Committee proposes that 5s. should be charged to the societies for a book of fifty.

These orders might possibly be of service to large societies covering a wide area, yet having only one central office and employing no agents. One such society, the Hearts of Oak Friendly Society, has in the past transacted practically all its business through the post, and might, therefore, derive some benefit from the issue of the proposed orders. It is not clear, however, that other approved societies—such, for instance, as those in connection with the large industrial insurance companies, the large friendly societies with many branches, or the small societies covering a very restricted area—will avail themselves to any extent of payment by post.

Payment is at present made by these societies by means of personal visitation, the agent in the case of the industrial insurance companies and the sick visitor in the case of the friendly societies being the channels through which the benefits are paid; and this personal visitation has such obvious advantages that it is hardly likely to be discarded.

Furthermore, if the orders are only supposed to be used when it is impossible to make payment in any other way, it does not seem that on the whole the proposed orders will be any less expensive than ordinary postal orders; rather the contrary.

For practically all benefit payments of approved societies, except maternity benefit, the cost of the requisite postal order would be 1d.; and, as it is proposed to charge 5s. for fifty of the new orders (presumably irrespective of amount), there would be no saving whatever in expense.

The advantage claimed for the new orders is, of course, the fact that they would contain particulars of the benefit in respect of which payment was being made, and would be countersigned by the payee. The post office, after payment, would return the orders to the respective societies, who would thus be in possession of the receipt of the insured person.

We are, however, of the opinion that the end desired would have been sufficiently attained if ordinary postal orders were bound together in books in precisely the same way as the proposed new orders, stamped, perhaps, with the name of the society and issued to societies when desired as payments to them out of the National Health Insurance Fund. The society would thus be charged with the amount at the outset, and the post office would be relieved of all trouble except that of returning the paid orders to the societies.

The question of adopting a modified postal order was, of course, fully considered by the Committee; but, in spite of their decision, we think that the adoption of some such method as above suggested would sufficiently have met the need.

### Sickness Benefit in Cases of "Debility."

A case of considerable interest to insured persons and of some importance to approved societies was heard recently in the Court of Appeal before Mr. Justice Ridley and Mr. Justice Rowlatt.

The facts are briefly as follows: An insured person (a woman) had sent a claim for sickness benefit to the approved society to which she belonged, and the medical certificate accompanying the claim was to this effect—"I hereby certify that she (the insured person) is suffering from debility, and is unable to work." The approved society refused to admit the claim on the ground that according to the Act and to its own rules sickness benefit is only payable during incapacity resulting from *a specific disease, or from bodily or mental disablement*. It was maintained that "debility" did not come under any of these heads, and the society requested definite information as to the specific disease from which the woman was suffering. The insured person thereupon sought the assistance of the County Court, asking for an injunction to restrain the approved society from refusing to accept the medical certificate and claiming three weeks' sickness benefit amounting to £1 2s. 6d. The society resisted the action, contending, *inter alia*, that the County Court had no jurisdiction in the matter, since the Act of 1911 clearly laid down in Section 67 that disputes between insured persons and approved societies must be settled in the manner prescribed by the rules of the latter. The rules of the society in this case referred all such disputes to a general meeting of delegates.

The County Court, however, gave judgment in favour of the insured person on the ground that the action of the society in refusing to admit the medical certificate was *ultra vires*, and that this fact brought the matter within the jurisdiction of the Court.

The society appealed against this decision, and the Court of Appeal decided in its favour. Mr. Justice Ridley, in giving judgment to this effect, stated that he did so mainly on the ground that the dispute was one regarding the construction of a rule, and, as such, must be decided according to the regulations of the society governing the settlement of disputes, the County Court being entirely unable to interfere. He furthermore stated that it was for the society, and no one else, to say whether "debility" was a sufficient description of a specific disease or of a mental or bodily disablement.

Indeed, any other decision would have caused approved societies to be subjected to innumerable petty and baseless actions every time they disputed the sufficiency of medical evidence of incapacity. Approved societies are, after all, not run for profit, but for the benefit of their members generally, and they may, therefore, be relied upon, on the whole, to act justly in such matters as these, and with due regard to the interests of all the members, including those claiming benefit. It should, of course, be noticed that the County Court had no jurisdiction because the matter was one of construction of rule, and the society was acting within its powers. Had it been otherwise the decision of the Court of Appeal would probably have been different. An instance of this occurred a short time back, when an approved society refused to acknowledge, in support of a claim for benefit, the medical certificate of a non-panel doctor. In this case it was decided that the action of the society was *ultra vires*, and it was, therefore, successfully sued in the Court.

With regard to the actual point at issue, whether or not "debility" is sufficient to justify a claim for sickness, there is a good deal to be said on both sides. The approved society, however, with the interests of other members to consider, was understandable in pressing for more definite information regarding the cause of incapacity. "Debility" is a general term of somewhat indefinite range, and while it is a sufficient cause of incapacity when it exists, it is, we imagine, a fairly good refuge for the malingerer.

In the great majority of cases of real debility it would not be difficult to be a little more precise regarding the nature of the illness. However this may be, the final decision on the matter now rests with approved societies, and each particular case will necessarily be treated on its merits.

### Is the Act Insolvent?

A good deal has been written lately, both in the daily papers and in the insurance press, regarding the alleged insolvency of the National Insurance scheme. The *Times* in particular celebrated the anniversary of the commencement of benefit payments under the Acts by publishing a long and pessimistic article endeavouring to prove conclusively that the present relation between contributions and benefits is unsound. This article and similar articles appearing in other papers have received considerable criticism from various quarters, and the matter is, therefore, at the present very much before public notice; the recent debate in the House of Commons has, of course, also added to the public interest in the subject. We have ourselves already touched upon the matter in these columns, and do not, therefore, propose to consider it at length in this issue. We have, however, introduced the subject in order to draw attention to a fact which is liable to be overlooked—namely, that the data at present obtainable is wholly insufficient as the basis for any reliable conclusions. We cannot do better, in illustration of this, than quote the words of Mr. Wedgwood Benn in answering a question on the subject in the House of Commons on February 16. The question referred in particular to approved societies for women, but the answer is applicable to all cases. Mr. Benn said: "It is impossible to say . . . whether the actuarial expectation has been exceeded until the age distribution . . . has been ascertained, and also until the accounts of the societies have been made up so as to show their actual experience for at least the first year of benefits. Information on the first head is now being forwarded by the societies to the offices of the Commissioners in connection with the crediting of reserve values. Full information on the second head will not be available until the accounts of the societies have been audited."

The importance of knowing the age distribution of the members before coming to any conclusion regarding the excess or otherwise of sickness claims will be apparent to all, and the fact that such information is not yet collated renders any attempt at definite conclusions futile. Furthermore, it would appear that it may be some time yet before anything like complete information regarding the first year's working is available. The societies are experiencing considerable and unexpected difficulties in compiling their statistics and keeping their accounts, and we therefore anticipate a somewhat long delay. As a typical example of the difficulties encountered it may be mentioned that several large approved societies find that they have in their possession a considerable number of fully stamped contribution cards, which in total run into some thousands, and yet which cannot be reconciled with any record in the societies' books. This, of course, means that the societies cannot at present claim credit for the contributions thus made to them, and the State reaps a temporary benefit. The matter will doubtless right itself in time, but the delay caused is likely to be considerable.

While, therefore, we are willing to admit that there are grave indications that point to a deficiency in certain societies as the result of the first valuation, we would lay stress on the fact that for the time being no definite opinion can be formed. The doubts that exist regarding the solvency of approved societies for women are doubtless the best grounded, but it must be remembered that this is not at all surprising in view of the fact that reliable data to form the basis for calculating the necessary contributions was practically non-existent.

